# Synthesis and Characterization of Hollow Mesoporous Silica Nanoparticles for Smart Corrosion Protection

**DOI:** 10.3390/nano8070478

**Published:** 2018-06-28

**Authors:** Cristina Zea, Jenifer Alcántara, Rosa Barranco-García, Manuel Morcillo, Daniel de la Fuente

**Affiliations:** 1 National Centre for Metallurgical Research (CENIM/CSIC), Avda. Gregorio del Amo 8, 28040 Madrid, Spain; czea@cenim.csic.es (C.Z.); j.alcantara@cenim.csic.es (J.A.); rbarranco@ictp.csic.es (R.B.-G.); morcillo@cenim.csic.es (M.M.); 2 Institute of Polymer Science and Technology (ICTP/CSIC), C/Juan de la Cierva 3, 28006 Madrid, Spain

**Keywords:** hollow mesoporous silica nanoparticles (HMSN), smart release, corrosion protection, anticorrosive coatings, phosphomolybdate

## Abstract

Different approaches have been considered for the development of smart anticorrosive coatings by the incorporation of nanocontainers loaded with corrosion inhibitors into the protective layer. Nanocontainers are designed to allow a controlled release of the inhibitor in response to an external stimulus, thus, achieving more efficient and more economical use of the active component. In this case, a pH change is a very interesting stimulus to trigger the release because corrosion processes cause local pH changes. To this end, a special focus has been placed on the use of mesoporous silica nanoparticles (MSN) as nanocontainers due to their interesting characteristics, such as larger surface area, versatile functionalisation, stability, etc. However, the use of hollow mesoporous silica nanoparticles (HMSN), with a large central hole combined with an external mesoporous silica shell, offers an additional advantage due to the higher loading capacity. In the present work, HMSN have been efficiently synthesised, loaded with sodium phosphomolybdate, as a non-toxic alternative to the use of chromates, and encapsulated by a layer of an oppositely charged polyelectrolyte, poly(diallyldimethylammonium chloride) (PDDA). The morphology and textural properties of the produced nanocapsules have been studied by different techniques (SEM/EDS, TEM/EDS, Brunauer–Emmett–Teller (BET) analysis method, ζ-potential). Finally, the releasing capacity and corrosion protection at different pH values have been studied, confirming the smart behaviour of the encapsulated loaded HMSN.

## 1. Introduction

Conventional anticorrosive organic coatings are mainly based on the incorporation of corrosion inhibitors, which are continuously released to protect metallic substrates. This constant leaching notably reduces the protection lifetime. In addition, anticorrosive coatings have been traditionally based on the use of Cr (VI) compounds as corrosion inhibitors [1]. However, it is known that chromates are highly toxic to both the environment and human health due to their carcinogenic effects [2]. For that reason, special attention is being paid nowadays to the development of new smart anticorrosive coating systems, with an improved efficiency that simultaneously increases the lifetime of the coating and reduces its impact on the environment. The improved efficiency is based on acting upon demand in the affected area in response to an external stimulus associated to coating damage (pH change, redox reactions, mechanical damage, etc.) [3,4,5,6,7].

In this sense, one of the most interesting studied approaches is to load active compounds into nanocontainers and then to incorporate them into the coating matrix [8]. A variety of different nanocapsules has been considered for meeting this objective: Ion-exchange pigments based on Layered Double Hidroxydes (LDH) [9,10,11], polyelectrolytes shells [12,13,14], nano- and microcapsules of different size, morphology, and composition (polymers, tubes, clays, inorganic compounds as silica, zirconia, titanium dioxide, etc.) [15,16,17,18,19]. However, among all types of nanocontainers, special focus has been centred on mesoporous silica nanoparticles (MSN) due to their high loading capacity, large surface area, stability, biocompatibility, controllable pore diameter, and easy surface functionalization based on the presence of Si-OH groups [20,21,22,23,24,25,26,27].

Alternatively, the use of hollow mesoporous silica nanoparticles (HMSN), with a large central hole and an external mesoporous silica shell, offers additional advantages due to the higher loading capacity, lower density, and larger specific area. Moreover, HMSN have been demonstrated to be very useful in many applications, such as enzyme encapsulation [28], drug transport and delivery [29], storage of volatile substances [30], light-emitting [31], etc. However, they have not been extensively studied as corrosion inhibitor nanocarriers, and only few studies have been carried out on it [32,33,34].

Zhao et al. synthesized HMSN with magnesium hydroxide precipitate in the shells, loading them with benzotriazole (BTA) as the corrosion inhibitor for aluminium substrates. They confirmed the release of BTA in acidic conditions and a good anticorrosive protection for aluminium [32].

Chen et al. also synthesized HMSN loaded with BTA as the corrosion inhibitor, achieving a pH-sensitive behaviour by installing nanovalves based on supramolecular complexes stalks. A sol-gel coating doped with both types of pH reactive nanocapsules showed a certain corrosion protection of AA2024 aluminium alloy substrate [33].

Similarly, Fu et al. loaded HMSN with caffeine molecules and installed bistable pseudorotaxanes as supramolecular nanovalves on the external surface of HMSN. The smart nanocontainers encapsulate caffeine molecules at a neutral pH and release the molecules either under acidic or alkaline conditions. A delay on the penetration rate of corrosive species and a successful repair of the damaged aluminium oxide layer were observed [34].

However, synthesis of the previously mentioned functionalised HMSN is complex and makes these methods difficult to up-scale and to apply in practice in the coatings industry. On the other hand, the use of BTA or caffeine molecules can offer acceptable results for the protection of aluminium or copper substrates, but they cannot be considered a real useful alternative to chromates for carbon steel protection [35,36,37].

To overcome these restrictions, in the present work, sodium phosphomolybdate, a non-toxic compound with very good anticorrosive behaviour for carbon steel protection [38], has been loaded into HMSN. To achieve a pH-responsive release, loaded HMSN have been encapsulated by a simple deposition of one layer of an oppositely charged polyelectrolyte, in our case poly(diallyldimethylammonium chloride) (PDDA). The morphology and textural properties of the produced HMSN have been studied by: Scanning Electron Microscopy/Energy Dispersive X-ray Spectrometry (SEM/EDS), Transmission Electron Microscopy/Energy Dispersive X-ray Spectrometry (TEM/EDS), N_2_ adsorption–desorption isotherms, and zeta potential (ζ-potential) measurements. Regarding the smart anticorrosive behaviour as a function of pH, the releasing capacity, as well as the polarization resistance, of the steel substrates exposed to aggressive solutions in the presence of smart nanocontainers has been studied.

## 2. Materials and Methods

### 2.1. Synthesis, Loading with Inhibitor, and Encapsulation of HMSN

**Figure 1:**
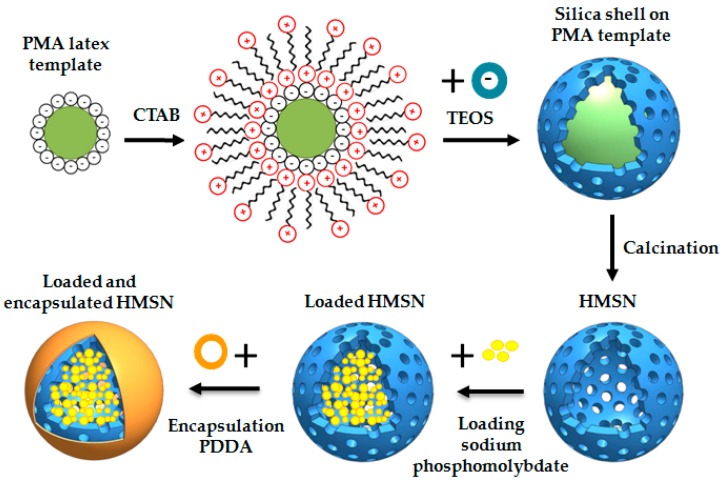
Synthesis of HMSN, loading with sodium phosphomolybdate and deposition of an external PDDA layer.

HMSN have been synthesised according to the methodology defined by Ge et al. [39]. A scheme of the process is shown in Figure 1.

According to the methodology described by Agrawal et al., Polystyrene-methyl acrylic (PMA) acid latex spheres were obtained by an emulsifier-free polymerization method [40]. These spheres will be used as core templates for HMSN. 50 mL of styrene and 5 mL of methyl-acrylic acid were added to 450 mL of deionized water and introduced into a 1000 mL two-necked flask. A mechanical stirrer, temperature controller, and N_2_ inlet were also mounted. Then, N_2_ was bubbled during 30 min to deoxygenate the mixture and the temperature was raised to 70 °C. When this temperature was reached and to initiate the polymerization, K_2_S_2_O_8_ (0.25 g in 10 mL of deionized water) solution was added. The polymerization process continued for 24 h and a steady dispersion of PMA in the water with ~10 wt % solid content was obtained. 50 mL of the previously obtained PMA dispersion was added to 150 mL of deionized water and kept under sonication for 5 min. Then, the pH of the solution was adjusted with ammonia aqueous solution (30 wt %) to pH = 10, and 20 mL of surfactant (5 wt % cetyltrimethylammonium bromide, CTAB) was also added and stirred for 1 h. Finally, 8 mL of tetraethylorthosilicate (TEOS) was added and stirred for 4 h at 25 °C, keeping the suspension at that temperature overnight without stirring. A membrane with a 0.1 μm pore size was used under vacuum to filter the obtained solution. The white powder retained in the filter was washed three times with deionized water. The white powder was calcinated to 550 °C at a heating rate of 1 °C·min^−1^ and kept at 550 °C for 4 h to get the desired structure with a central hole and an external mesoporous silica shell to serve as the smart nanocontainer.

After preparation of the empty HMSN as described above, the loading stage was carried out as follows: 500 mL of 0.01 M Mo_12_Na_3_O_40_P solution was prepared and the pH was adjusted to 2.3. It is known that loading is improved at acidic pH values and the optimum value of 2.3 was established in previous studies [20,25]. Then, 1 g of the prepared empty HMSN was added and kept under continuous stirring for 8 hours. A filter membrane with a 0.1 μm pore size was used under vacuum to filter the obtained solution. Finally, a layer of a positively charged PDDA polyelectrolyte was deposited on the external mesoporous silica shell by immersion of the loaded HMSN. To efficiently deposit polyelectrolytes on a surface, a high ionic strength is needed. Otherwise, an electrostatic barrier forms between the already adsorbed layers and the molecules from the bulk solution, hindering the deposition of new additional macromolecules and stopping, in that case, the adsorption. For that reason, NaCl or similar salts solutions are generally added for easy deposition of polyelectrolytes layers. Therefore, in our case, 0.75 g of PDDA were dissolved in 25 mL of 0.5 M NaCl solution. The mixture was kept under stirring for 15 min. Furthermore, 0.35 g of loaded HMSN was firstly added to 25 mL of 0.5 M NaCl and then incorporated into the PDDA solution. A membrane with a 0.1 μm pore size was used under vacuum to filter the obtained solution. The retained HMSN, loaded and encapsulated with PDDA, were washed three times with deionized water. Finally, they were dried at 45 °C for 2 h.

### 2.2. Characterization Study

Both Scanning and Transmission Electron Microscopies with Energy Dispersive X-ray Spectrometry (SEM/EDS and TEM/EDS) were used to observe and analyse the HMSN at the different stages: Core templates, before and after calcination, after loading, and after PDDA deposition. Hitachi S4800 (Hitachi, Tokyo, Japan) and Philips Tecnai 20T (Thermo Fisher Scientific, Waltham, MA, USA) were used for SEM and TEM observations, respectively. An Oxford Inca microanalysis system was coupled to both microscopes. Before observation, the particles were spread in acetone, kept under sonication for 5 min, and placed onto an iron or copper grid in the case of SEM and TEM, respectively.

N_2_ adsorption-desorption isotherms at 77 K were used to obtain the specific surface area (S_BET_), average pore diameter (Øp), and pore volume (Vp) at the different stages. The equipment used was a Micromeritics TRISTAR 3000 (Micromeritics, Norcross, GA, USA). The Brunauer-Emmett-Teller (BET) method was used to calculate the S_BET_ from the adsorption data in the low pressure range (0.05 ≤ P/P_0_ ≤ 0.2). The average pore diameter and pore volume were determined from the N_2_ adsorption branch by the Barret-Joyner-Halenda (BJH) method, defining the thickness of the adsorbed N_2_ layer by means of the Harkins and Jura equation.

The zeta potential (ζ-potential) was measured by a Zetasizer nanoZ (Malvern Instruments Ltd., Malvern, UK) at 25 °C with the Smoluchowski approximation. 10 mg of nanoparticles at each stage, before and after calcination, after loading, and after PDDA deposition, was dispersed in 50 mL of deionised water. To obtain the ζ-potential average, three different sample dispersions were measured also by triplicate.

### 2.3. Evaluation of Inhibitor Release as a Function of pH

Fifty milligrams of loaded and encapsulated HMSN were added to 25 mL of deionized water at six pH values (1, 3, 5, 7, 9, and 13). The pH was adjusted by means of the addition of HCl or NaOH solution as applicable, and it was measured by a standard pH electrode (Crison 52 03). Before filtering, the samples were kept under continuous stirring for 30 min. Next, a membrane with a 0.1 μm pore size was used under vacuum to filter the solutions. The aqueous extract was used to determine by inductively coupled plasma optical emission spectrometry (ICP-OES) the Mo content. The equipment used was a PerkinElmer 4300 DV (PerkinElmer, Waltham, MA, USA) after calibration with a standard Mo solution.

### 2.4. Evaluation of Smart Anticorrosive Behaviour

Polarization Resistance (Rp) measurements were carried out in a classic three-electrode cell consisting of an Ag/AgCl reference electrode, a stainless steel counter electrode, and a carbon steel specimen as a working electrode in the horizontal position, with a working area of 6.16 cm^2^. The carbon steel working electrodes were ground with SiC papers to a 600-grit-finish and cleaned with ethanol in an ultrasonic bath for 5 min. After that, measurements were carried out at room temperature using a potentiostat/galvanostat (AutoLab EcoChemie PGSTAT30) (Metrohm AG, Herisau, Switzerland) equipped with NOVA 1.7 software. The scanning range was ±20 mV vs. Open Circuit Potential (OCP) at a scanning rate of 0.5 mV/s. The electrolyte used was 10 mM Na_2_SO_4_ solution without and with the addition of 2 mg/mL of loaded and encapsulated HMSN at four different pH values (7, 9, 11, and 13). Measurements were carried out after 30 min and 24 h of exposure to the electrolyte.

## 3. Results and Discussion

**Figure 2:**
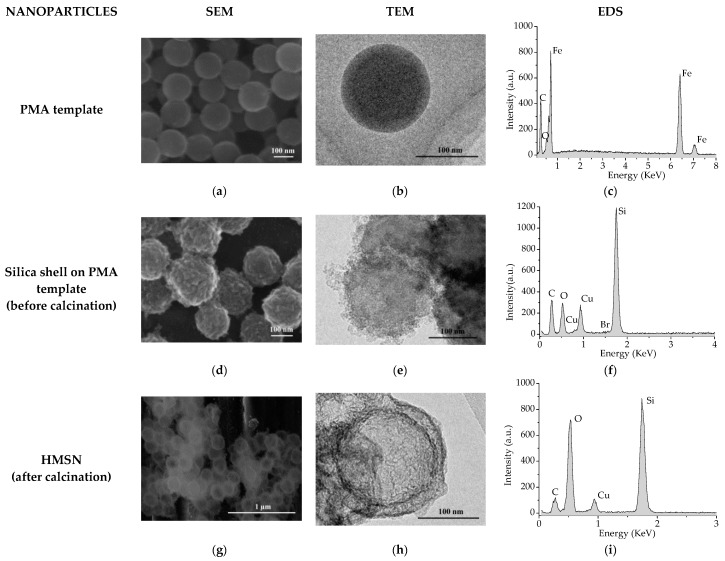
SEM and TEM images and EDS spectrums obtained: On PMA templates (**a**–**c**); on particles before calcination (**d**–**f**,); and after calcination (**g**–**i**), respectively.

SEM, TEM, and EDS analysis of PMA templates are presented in Figure 2a–c, respectively. As can be seen in both the SEM and TEM images, PMA spheres to be used as core templates were effectively synthesised as monodisperse spherical nanoparticles with a diameter of around 150 nm. The presence of Fe in the EDS analysis derives from the carbon steel sample used as the metallic substrate for HMSN deposition while the presence of C and O corresponds to PMA.

Figure 2d,e show the SEM and TEM images of the nanoparticles, respectively, before the calcination stage, but after the building-up of the silica shell on the PMA templates. Silica shell still containing the surfactant can be observed around the core templates and is confirmed by the detection of Si, O, and Br in the EDS analysis carried out (Figure 2f). In this case, the presence of Cu derives from the grid used for particle deposition.

After calcination, a central cavity is formed due to removal of the core template, as well as an outer shell of mesoporous silica, formed because of the removal of the surfactant template. As can be seen on both the SEM and TEM images presented in Figure 2g,h, respectively, the existence of a hollow shell (150 nm in diameter) and a silica shell (25 nm thickness) has been confirmed. As a result, HMSN with a full diameter of around 200 nm have been successfully synthesised and could be, as expected, a feasible candidate to act as nanocontainers for corrosion inhibitors.

**Figure 3:**
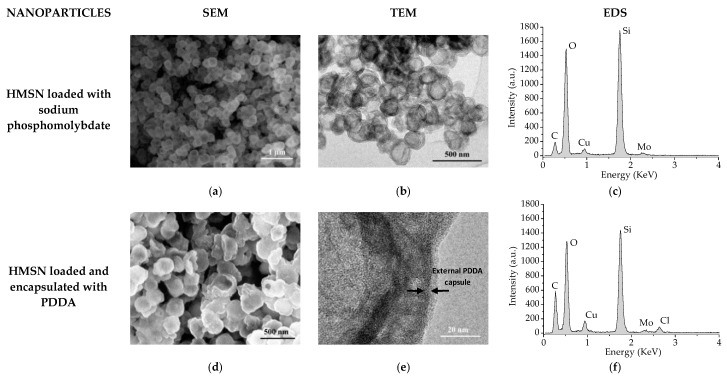
SEM, TEM, and EDS images and spectrums obtained on HMSN loaded with sodium phosphomolybdate before (**a**–**c**) and after encapsulation with PDDA (**d**–**f**).

In Figure 3a–c, SEM, TEM, and EDS analysis of HMSN after loading with sodium phosphomolybdate are shown. As can be observed, the morphology of the HMSN does not show any relevant change after the loading stage. On the other hand, detection of Mo in the EDS spectrum indicates that the inhibitor has been successfully loaded into the HMSN structure.

SEM, TEM, and EDS analysis of HMSN after encapsulation with PDDA are presented in Figure 3d–f, respectively. As can be seen in the TEM image (Figure 3e), the presence of an external capsule, deposited on the silica shell, of 5–15 nm in thickness can be intuited. Additionally, the presence of Mo (Figure 3f) clearly indicates that phosphomolybdate remains inside the HMSN after the PDDA layer deposition stage.

**Figure 4:**
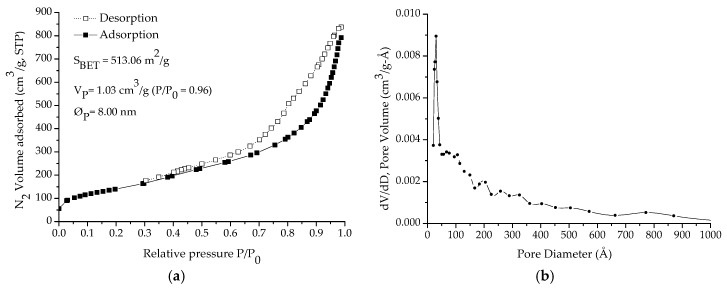

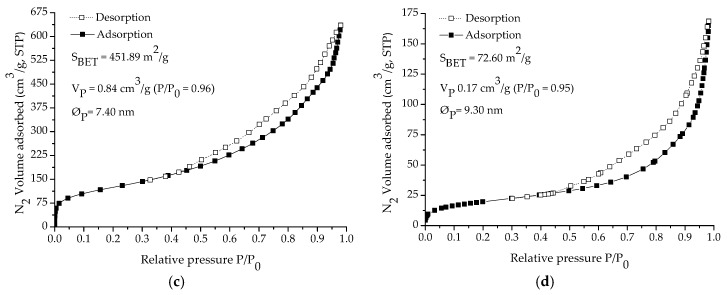
N_2_ adsorption-desorption isotherm (**a**) and pore size distribution (**b**) of HMSN after calcination. N_2_ adsorption-desorption isotherms after loading with inhibitor (**c**) and after encapsulation with PDDA (**d**). S_BET_: Specific surface area, V_p_: Pore volume, and φ_p_: Pore diameter.

The N_2_ adsorption-desorption isotherms obtained on HMSN after calcination, and after loading with the inhibitor and after encapsulation with PDDA, are presented in Figure 4a,c,d respectively. In addition, the pore size distribution obtained on HMSN after calcination is presented in Figure 4b. As expected, after calcination, the isotherms corresponded to type IV with a hysteresis cycle (type H2) above P/Po~0.4 and an asymptotic closure at that P/Po value, which is characteristic of mesoporous materials. As can be seen, both S_BET_ and V_p_ decreased after the inhibitor loading stage. Then, as previously indicated by the presence of Mo in the EDS spectrums (Figure 3c,f), the BJH study validates the successful loading of HMSN with sodium phosphomolybdate. Finally, the presence of the external PDDA layer causes, as expected, a noteworthy additional reduction of both S_BET_ (86%) and V_p_ (83%), thus, confirming the successful encapsulation.

**Figure 5:**
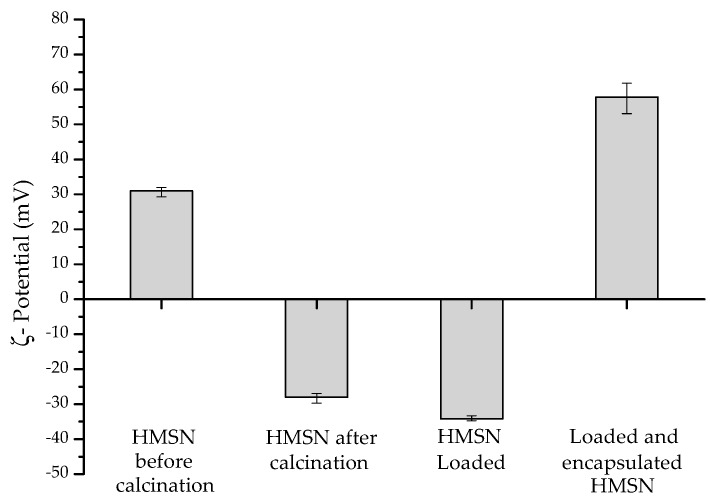
ζ-Potential values of HMSN at each different stage.

In Figure 5, the average ζ-potential values of the HMSN at each different stage are presented. As can be observed, nanoparticles of the silica shell on the PMA template (before calcination) present a positive zeta potential (+31.0 mV) due to the still presence of the surfactant structure in the pores. However, after calcination and removal of both the surfactant molecules and core template, the ζ-potential of the empty HMSN shifted to negative values (−28 mV). After loading the inhibitor, a minor decrease (up to −34.2 mV) was measured. In contrast, deposition of the PDDA layer led to a noteworthy change to positive values (+57.8 mV). Therefore, successful encapsulation of the active inhibitor inside the HMSN structure was confirmed, as well as the presence of the external PDDA layer deposited on the silica shell surface.

**Figure 6:**
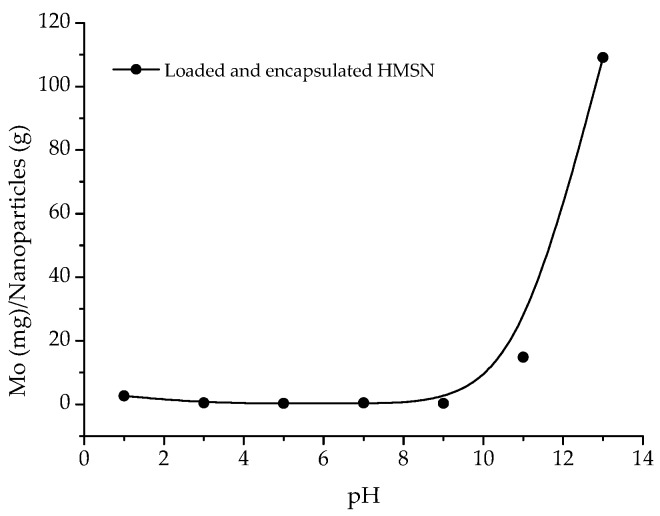
Inhibitor release as a function of pH.

Results of the inhibitor release as a function of the pH are presented in Figure 6. As can be observed, from pH 3 to pH 9 the inhibitor release is almost completely prevented. On the other hand, from pH = 9, a progressive increase of inhibitor discharge was observed. At pH = 13 it may be considered that the full amount of inhibitor loaded into the nanoparticles has been completely released. At this pH neither the PDDA layer nor the silica are stable. As release takes place under alkaline conditions, but loading is favoured under acidic ones, the reversibility of the process is strongly hindered, thus, allowing that, once released, the corrosion inhibitor can act in the affected area and the discharge is only stopped when the pH decreases sufficiently. Therefore, the smart release of the inhibitor from HMSN as a function of pH has been clearly confirmed.

**Figure 7:**
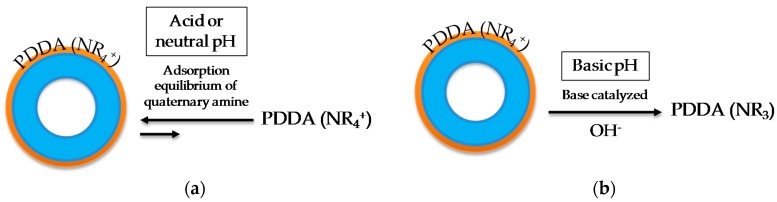
Behaviour of deposited PDDA layers as a function of pH: (**a**) Acid or neutral pH and (**b**) basic pH.

These results agree with previous research on this topic. For example, Sukhorukov et al. reported that the PDDA layer is usually impermeable in a certain pH range [41]. Deposited PDDA layers have no H^+^ to yield and are not affected by OH^-^ ions in solution up to a certain concentration (Figure 7a). As a result, they stay charged regardless of the external pH and exhibit almost no variation in electroosmotic flow [42,43]. Nevertheless, quaternary amine-based layers, such as PDDA, are not stable under alkaline conditions, and a drifting electroosmotic flow has been verified at pH > 8 [44,45,46]. At exceptionally alkaline pH values they may form a hydroxide that can undergo an E2 Hofmann elimination reaction (Figure 7b) [47].

**Figure 8:**
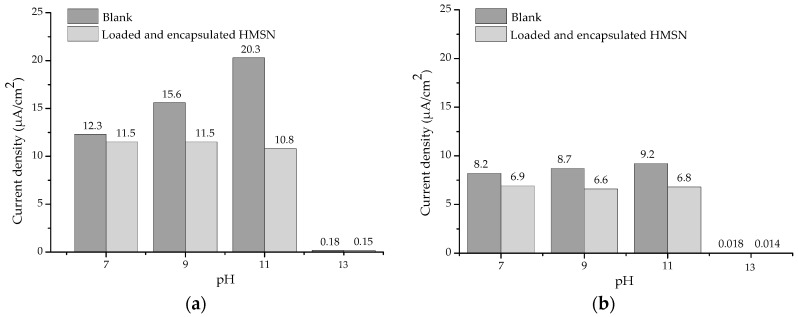
Current density as a function of pH obtained from Rp measurements: (**a**) After 30 min of exposure and (**b**) after 24 h of exposure.

Regarding the smart anticorrosive behaviour as a function of pH, the current densities obtained from Rp measurements performed on carbon steel substrates after 30 min and 24 h of exposure to an aggressive electrolyte (10 mM Na_2_SO_4_ solution) at different pH values, without and with the addition of loaded and encapsulated HMSN are presented in Figure 8.

As can be seen, after 30 min of exposure the presence of nanocontainers does not cause a significant reduction of the current density at pH 7. As explained before, at this pH value an almost closed state of the nanocontainers is expected. However, at pH 9, and especially at pH 11, a clear reduction by 26% and 47%, respectively, is observed due to the desirable release of the corrosion inhibitor. At pH 13, very low current densities were measured in both cases, as was expected, due to the passivity stage of carbon steel at this pH according to the Pourbaix diagram. Even after 24 h of exposure, a reduction by 24% and 26% at pH 9 and pH 11, respectively, was still measured, clearly indicating the beneficial effect and the smart anticorrosive protection of the developed HMSN loaded with phosphomolybdate and encapsulated by an external PDDA layer.

## 4. Conclusions

Hollow mesoporous silica nanoparticles have been effectively synthesised, then loaded with sodium phosphomolybdate, as a non-toxic inhibitor alternative, and encapsulated by an external layer of poly(diallyldimethylammonium chloride) (PDDA). The presence of this external layer allows a pH-controlled release of the inhibitor, almost completely avoiding any discharge in the pH 3–9 range. Then, a progressive raise in the amount released at pH > 9 and a complete release at pH = 13 has been confirmed. The smart release of the corrosion inhibitor improves the anticorrosive protection of carbon steel substrates at a certain, basic pH. Therefore, the developed pH-dependent nanocapsules could be considered as a possible future alternative to the use of toxic chromates in anticorrosive organic coatings.
